# The Roles of Aquaporins in Plant Stress Responses

**DOI:** 10.3390/jdb4010009

**Published:** 2016-02-04

**Authors:** Zunaira Afzal, T. C. Howton, Yali Sun, M. Shahid Mukhtar

**Affiliations:** 1 Department of Biology, University of Alabama at Birmingham, Birmingham, AL 35294, USA; zunaira1@uab.edu (Z.A.); tchowton@uab.edu (T.C.H.); yalisun@uab.edu (Y.S.); 2 Nutrition Obesity Research Center, University of Alabama at Birmingham, Birmingham, AL 35294, USA

**Keywords:** aquaporins, abiotic stress, biotic stress, drought, salinity, cold stress, osmotic stress, nutrient homeostasis

## Abstract

Aquaporins are membrane channel proteins ubiquitously present in all kingdoms of life. Although aquaporins were originally discovered as water channels, their roles in the transport of small neutral solutes, gasses, and metal ions are now well established. Plants contain the largest number and greatest diversity of aquaporin homologs with diverse subcellular localization patterns, gating properties, and solute specificity. The roles of aquaporins in physiological functions throughout plant growth and development are well known. As an integral regulator of plant–water relations, they are presumed to play an important role in plant defense responses against biotic and abiotic stressors. This review highlights involvement of various aquaporin homologs in plant stress responses against a variety of environmental stresses that disturb plant cell osmotic balance and nutrient homeostasis.

## 1. Background and Discovery of Aquaporins

Water, quite distinctly, is the universal solvent paramount for all living cells. The transportation of water within an organism or between the organism and its environment is crucial to accomplishing all fundamental life processes. Since the discovery of the lipid bilayer in the 1920s, the flow of water across cells and subcellular compartments was assumed to be carried out by simple diffusion across biological membranes. However, the diffusion of water across membranes occurs too slowly to account for particular physiological processes such as secretory pathways in mammals and stomatal aperture regulation in plants. These processes require a rapid, reversible flow of large volumes of water across membranes. Moreover, subcellular membranes within the same cell exhibit remarkably different levels of water permeability that cannot be justified solely by simple diffusion [1].

**Figure 1:**
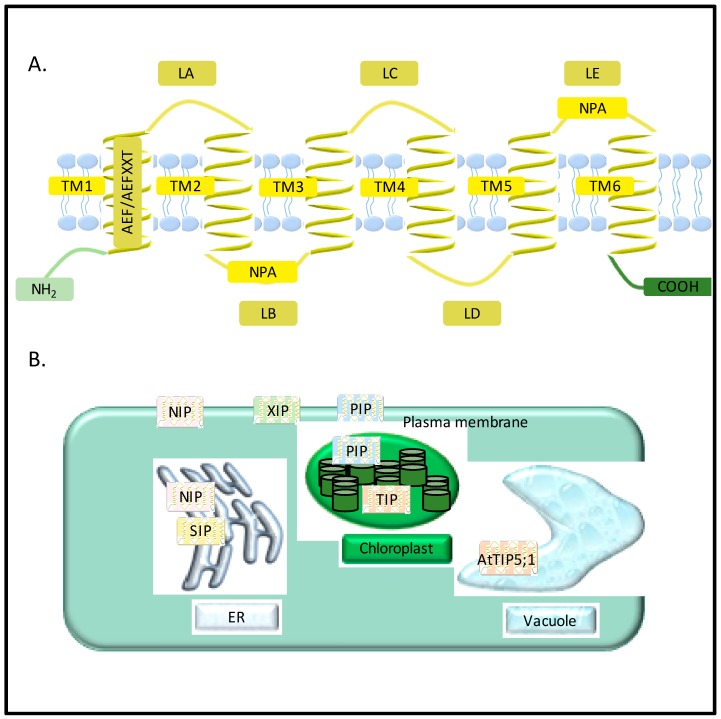
General structure and localization of aquaporins. (**A**) Major intrinsic protein (MIP)monomer includes six alpha helical transmembrane helices (TM1–TM6), five inter-helical loops (LA-LE), an AEF(Ala-Glu-Phe) or AEFXXT motif in the N-terminal domain and two highly conserved NPA (Asp-Pro-Ala) motifs; (**B**) General localization of aquaporins. Plasma membrane intrinsic proteins (PIPs), nodulin-26 like intrinsic proteins (NIPs), and uncategorized X intrinsic proteins (XIPs) are generally localized to the plasma membrane, and expressed on the entire cell surface. Small basic intrinsic proteins (SIPs) and some NIPs have been found localizing to endoplasmic reticulum (ER). Tonoplast intrinsic proteins (TIPs) are localized to tonoplast, the membrane of vacuole. Some PIPs and TIPs have been predicted to localized to the inner envelop and thylakoids, while AtTIP5;1 has been found to be located to tonoplast.

To account for these processes, the transport of water across biological membranes through specialized pores rather than by simple diffusion was first proposed by Koefoed-Johnsen *et al.* in 1953 [2] and then confirmed by Macey *et al.* in 1970 [3]. The proteinaceous nature of this pore came to light in the late 1980s with the advent of CHIP28, a highly abundant, channel-forming, integral membrane protein of 28-kDa isolated from human erythrocytes [4]. The molecular identity of the first water channel protein, aquaporin 1 (AQP1) was established by Agre and coworkers in 1992 based upon its ability to dramatically increase the water permeability of *Xenopus oocytes* expressing the *CHIP28* gene [5]. Although the first aquaporin member, Nodulin-26, GmNOD26 was identified in soybeans as early as 1987 [6], Wayne *et al.* in 1990 suggested the presence of pertinacious pores in plant membranes even before its discovery in humans. Quite uniquely, the existence of water channels in plants was not clearly hypothesized until 1993 when Maurel *et al.* demonstrated the functional expression of the first plant aquaporin, Arabidopsis tonoplast intrinsic protein homolog (AtTIP1;1), in *Xenopus. laevis* oocytes [7] (Figure 1).

Aquaporins belong to a highly conserved super family of membrane proteins known as major intrinsic protein (MIP). MIPs are ubiquitously present in all living organisms except thermophilic Archaea and intracellular bacteria [8] (Figure 1). Currently, aquaporins are recognized as the most abundant transmembrane transporters of substrates like glycerol, urea, CO_2_, NH_3_, metalloids, and reactive oxygen species (ROS) in addition to water. [9]. Due to their sedentary nature, the absence of a specialized circulatory system, and a large number of intracellular compartments, plants exhibit a great necessity for fine-tuned water regulation to adapt to environmental fluctuations. Presently, 35 aquaporin encoding genes in *Arabidopsis thaliana* (thereafter Arabidopsis) [10], 31 in maize [11], 33 in rice [12], 34 in sweet orange [13], 47 in tomato [14], 55 in *Populus trichocarpa* [15], 66 in soybean [16], 50 in banana [17], 71 in cotton [18], and 41 in sorghum [19] have been identified. This review will explicate the roles of the seven subfamilies of aquaporins in drought and salinity responses, stomatal regulation, chilling responses, nutrient uptake and transport during growth and development, and biotic stress responses.

## 2. Diversity of Aquaporins in Plants

Presently, more than 800 MIPs [20,21] have been identified in bacteria [22], yeast [23], protozoa [24], archaea [25], insects [26] mammals [5,27] and plants [6]. Based on substrate specificity and protein sequence similarities, these 800 MIPs have been classified into three main subfamilies: (1) AQPs (aquaporins) involved in water and ion transport; (2) GLPs (glycerol-facilitators) permeable to glycerol and neutral molecules; and (3) GLAs (aquaglyceroporins) permeable to both water and glycerol. [20]. Unicellular organisms such as bacteria or yeast present the least MIP diversity, typically possessing only one or two aquaporin encoding genes. Plants do not have GLP orthologs [28], but they do exhibit the greatest diversity of ubiquitously localized AQPs [29,30] in comparison to mammals with 13 aquaporin isoforms (AQP0-12). Mammalian orthologs are primarily restricted to secretory glands [31] and fluid-conducting organs. Owing to the abundance and diversity of aquaporins in plants, MIPs are usually assimilated in a broader sense as aquaporins in literature (Figure 1).

Aquaporins include seven subfamilies categorized according to their intracellular locations and sequence similarities: the plasma membrane intrinsic proteins (PIPs), tonoplast intrinsic proteins (TIPs), NOD26-like intrinsic proteins (NIPs) and small, basic intrinsic proteins (SIPs), the GlpF-like intrinsic proteins (GIPs), hybrid intrinsic protein (HIP), and the uncategorized X intrinsic protein (XIP) [32] (Figure 1). Each subfamily could be further divided into different subgroups. For example, PIPs have been divided into two subgroups, PIP1 and PIP2. Each subgroup is then again divided to different isoforms, such as PIP1;1 and PIP1;2, which have specialized locations and functions (Figure 1).

The common characteristics that all MIPs share include six alpha-helical transmembrane helices (TM-1-TM-6), five inter-helical loops (LA-LE) [33] an AEF(Ala-Glu-Phe) or AEFXXT motif in the *N*-termial domain, and two highly conserved NPA (Asp-Pro-Ala) motifs (the “NPA box”) (Figure 1). Transport substrates, expression pattern, different levels of modification, regulation, and intracellular localizations contribute to the differences between the subfamilies of plant aquaporins. PIPs, NIPs, and XIPs are generally localized to the plasma membrane and expressed on the entire cell surface, while TIPs are localized to the tonoplast, the membrane of the vacuole. For most plant aquaporins, localization on endoplasmic reticulum (ER) can be observed during the processes of post-transcription, translation, and modification (Figure 1). However, SIPs and some NIPs have been found to localize to the ER [9], although the mechanism of targeting and their cellular functions are still not clear. SIPs tagged with green fluorescent protein were transiently expressed in Arabidopsis cells, showing the ER subcellular localization [34]. SIP1;1 and SIP1;2 may function as water channels in the ER, while SIP2;1 might act as an ER channel for other small molecules or ions [34]. Based on proteomic analyses in Arabidopsis, some PIPs and TIPs have been predicted to localized to the inner envelop and thylakoids [9], while it has also been found that AtTIP5;1 is located to tonoplast [35] (Figure 1).

Another difference between various MIP subfamilies is their substrate selectivity. Besides their function as water transporters, MIPs have been reported to transport atypical substrates, including ammonia, antimony, arsenite, boron, carbon dioxide, formamide, glycerol, hydrogen peroxide (H_2_O_2_), lactic acid, silicon, and urea. Two factors that contribute to their substrate selectivity are the conserved NPA motifs and amino acid residues including the ar/R (aromatic/arginine) region. The conserved NPA motifs contribute to the selectivity for water molecules [32], which are highly conserved in plant PIPs and TIPs, while alternative motifs have been found only in the NIP or SIP groups [36,37]. PIPs function as the transporters of water, glycerol, H_2_O_2_, carbon dioxide, and urea. PIP aquaporins are also involved in abiotic stress responses. However, several studies demonstrated altered responses of PIPs and TIPs family members upon salt, drought, or cold stresses [32]. The main role of TIPs has been described in the permeability of water. The water permeability function of plant aquaporins was first demonstrated in a TIP from Arabidopsis. Further studies have shown that TIPs may control water exchange between cytosolic and vacuolar compartments, which implies that they have a role in regulating cell turgor. Besides its water permeability function, TIPs also play roles in glycerol, urea, and ammonia transport and abiotic stress response. Specific TIP isoforms of rice and maize or Arabidopsis also show differential responses to water stress, salt, and cold stress. NIPs are able to transport water, glycerol, ammonia, silicic acid, and other solutes between plant and bacterial symbionts. Compared to PIPs and TIPs, NIPs have less water transport activity, but higher permeability to small organic molecules and mineral nutrients. The functions of NIPs include transporting beneficial and toxic metal molecules [38,39]. For example, OsNIP2;1 not only functions as a silicon influx transporter but is also involved in selenite uptake in rice [40]. SIPs have moderate water transport activity and may also function in original pore conformation. XIPs work as multifunctional channels permeable to water, metalloids and ROS. For example, *PtXIP2;1* exhibited a differential expression in leaves and stems under the stress of drought, salicylic acid, or wounding [41].

## 3. Roles of Aquaporins in Plant–Water Relations

**Figure 2:**
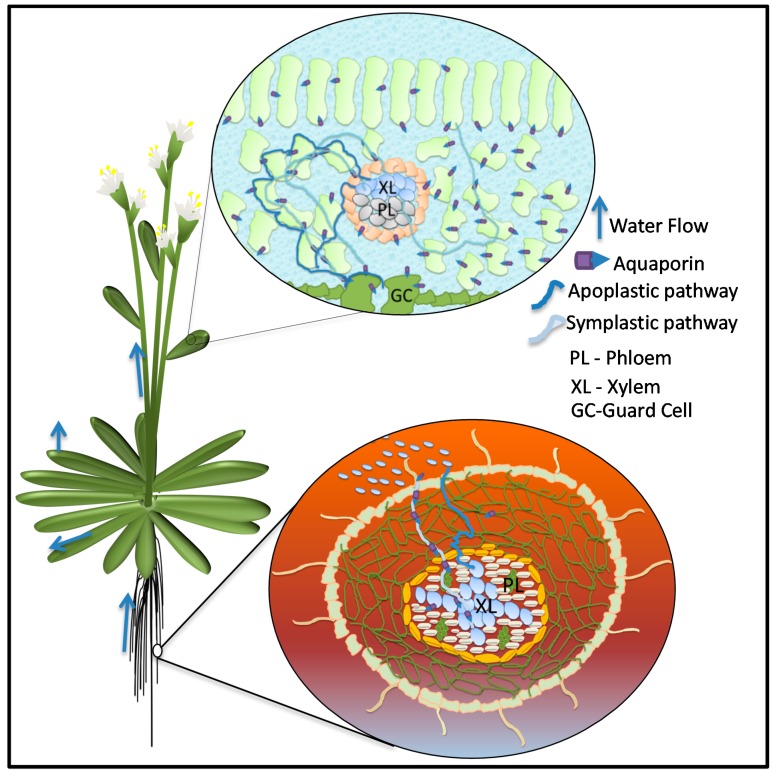
Water flow in plant from roots to aerial parts. Leaf cross-section shows flow of water through apoplastic and symplastic water pathways. In apoplastic pathway, water flows across the cells through cell wall. In symplastic pathway, water moves through cytoplasm and cellular membranes. Aquaporins are involved in symplastic path facilitating transmembrane water flow across cells and subcellular compartments.

Due to their autotrophic nature, plants require light and CO_2_ from their aerial environment culminated with water and nutrients from soil to carry out photosynthesis. A constant water flow starting from the absorption of water from soil to its distribution throughout the plant body and evaporation in the atmosphere is crucial for carrying out all the physiological activities of the plant and managing stress imposed by fluctuating environment. Plant–water relations result in establishing a soil-plant-atmosphere continuum [42]. Although water is a key element of all physiological processes, the plant, for its growth and metabolism, uses only a small fraction. The remaining 99.5% is lost during transpiration [43]. In order to fix one kilogram of carbon during photosynthesis, plants transport several hundred kilograms of water [44]. This bulk flow of water through plants can take three different routes: the apoplastic route along cell wall structure, the symplastic route from cell to cell through the plasmodesmata, and the transcellular path across the cellular membranes [45] (Figure 2).

Long-distance bulk flow of water during transpiration and sugar transport takes the apoplastic route through vascular bundles and is generally not limited by membrane barriers [46]. Water ascends through the xylem and phloem by capillary action driven by the gradient of water potential (ΔΨ) in which water moves from a region higher Ψ to a region of lower Ψ. In the light phase, stomatal opening leads to evaporation that causes a decreases in leaf Ψ that pulls water by creating tension in the xylem vessels resulting in the movement of water from the soil through the roots [42,47] (Figure 2).

Short-distance non-vascular water transport across cellular membranes is crucial to maintaining intracellular hydrostatic pressure (turgor) and cell water homeostasis at each level of organization. This includes (1) at the cell level: cellular/subcellular metabolic reactions, cell division, differentiation and elongation; (2) at the tissue level: stomatal movement; and (3) at the organ level: leaf petiole movement and maintenance of upright status of whole plant all processes depend upon cell water relations. Aquaporins as transmembrane water and solute transporter channels could be speculated as potential regulators of plant cell water relations that reflect their key roles in plant cell osmoregulation [48], root hydraulic conductivity (*L*p_r_), leaf hydraulic conductivity [49], transpiration [50], and cell elongation [51] (Figure 2).

Maintenance of the cell’s osmotic potential under stress conditions such as pathogen infection, drought, flooding, salinity, high or low temperatures, and biotic stresses is a major challenge for plant growth and development. The majority of abiotic stress conditions directly impact plant water relations and stimulate an array of complicated cellular and physiological responses that lead to turning on plant water-saving strategies such as stomatal closure to cut off water loss during transpiration. As a tradeoff, water-saving photosynthetic activity is decreased due to the unavailability of CO_2_, ultimately leading to decreased plant biomass production. Consequently, a dire need exists to understand the underlying mechanisms which control plant–water relations in relation to photosynthesis and their response to biotic and abiotic stresses [52]. Aquaporins, as vital regulators of plant–water relations, are potential targets in developing stress resistant crop plants. Their significance in all facets of plant growth and development is well-established, but the mechanistic pathways behind their roles under plant defense responses remains to be elucidated [9,32]. Numerous comparative transcriptome studies under various abiotic stress conditions have shown a differential response of different aquaporin homologs in diverse plant tissues.

Reverse genetic approaches have also been extensively used to fine-tune the role of different aquaporin encoding genes in response to diverse environmental stresses. However, the presence of a large number of diverse aquaporins, integrated complex expression patterns, and technique limitations for measuring accurate solute and water movement across transmembrane aquaporin channels are major hurdles in establishing their conclusive roles in plant growth and survival under abiotic and biotic stresses [46].

## 4. Plant Aquaporins in Water Stress

Based on conclusive evidence, it is widely accepted that, in most plant species, water uptake and transcellular water flow in roots are largely mediated by PIPs and TIPs. These are the most abundant aquaporins in the plasma membrane and tonoplast of the plant cells, respectively [53]. Comparative transcriptome studies revealed differential expression of multiple aquaporin homologs in response to drought stress suggesting definite roles in stress responses. Induced expression of *AtPIP2;3* under drought stress conditions is one of the earliest evidence of a drought responsive aquaporin [54]. In 2005, Alexandersson *et al.* monitored the expression of all 35 aquaporin homologs in Arabidopsis in response to drought stress alone and found that most *PIP* and some *TIP* genes have high levels of expression, while *NIP* genes have very low expression [55]. The authors also showed that all PIP genes are down-regulated in drought stress response in leaves except *AtPIP1;4* and *AtPIP2;5*, which are up-regulated. Moreover, *AtPIP2;6* and *AtSIP1;1* are constitutively expressed and are not significantly affected by the drought stress [55]. Expression of *AtPIP2;5* was also significantly up-regulated in leaves in response to a combination of drought and heat stresses [56]. Consistent with these results, several other studies in Arabidopsis have shown that among all subfamilies of aquaporins PIPs are most responsive to drought stress and most of them undergo a transcriptional down-regulation. Only a few genes were found to be up-regulated [57,58]. All of these *PIP* genes that are down-regulated in response to drought are highly expressed in the roots. Strong down-regulation of *PIP genes* transcription under drought stress was also observed in the roots and twigs of olive plants [59], in the roots of tobacco [60], and in the fruits of peach [61]. Moreover, most of these drought responsive transcriptional changes are conserved among different Arabidopsis accessions [57]. Distinctly, in many other plant species, differential responses by the same aquaporin homologs have been seen among different cultivars of the same plant species. For example, in the grapevines the expression of *VvPIP1;1* in the root was up-regulated by drought stress in an anisohydric cultivar but not in an isohydric cultivar [62]. In another experiment, expression of the *VvPIP2;1* gene was down-regulated under drought conditions [63]. Therefore, contrasting results have been reported between similar aquaporin homologs within the same species under drought stress. One transcriptome comparison of a field-grown cotton (*Gossypium. hirsutum*) under normal and drought stress conditions revealed that two highly homologous genes, *PIP1;3* and *PIP1;1* have contrasting expression patterns in leaves and roots [18] indicating that their roles in drought stress response differs despite their structural similarity. In addition to water deficiency dehydration caused by other environmental stimuli exhibited differential responses in some species. For example, osmotic stress induced by 10% polyethylene glycol (PEG) in rice revealed no effect on *OsPIP1;3,* but expression of *OsPIP1;1* and *OsPIP1;2* was up-regulated [64]. Contrastingly, expression of *OsPIP1;1* was down-regulated in osmotic stress administrated by manitol [65] and also by drought stress [66]. Similar alterations were seen in reddish aquaporins in response to salt, PEG, and mannitol induced osmotic stress [67]. Recently, characterization of *GoPIP1* from a legume forage *Galega orientalis* showed its association with drought tolerance. In contrast to rice and reddish, the transcripts levels of *GoPIP1* increased significantly in roots upon exposure to the osmotic stress imposed by both high NaCl concentration and PEG. Overexpression of this gene in transgenic Arabidopsis made the plants more vulnerable to drought stress but not to salinity stress [68]. Stress levels also lead to differential responses, for example, in grapevine leaves, moderate drought stress led to a significant down regulation of the five PIP genes investigated, but prolonged stress increased their expression levels [69]. Water transport by aquaporins is also coupled with diurnal rhythms. Diurnal expression of PIPs in response to different intensities of drought was investigated in *Fragaria vesca*. Researchers found that most of the PIPs are down-regulated in roots and the expression of *FvPIP1;1* and *FvPIP2;1* was strongly correlated to the decrease in substrate moisture contents. In leaves, the amplitude of diurnal aquaporin expression was down-regulated in response to drought [70]. Responses of aquaporins to drought stress also vary based on plant symbiosis. In a study of *Phaseolus vulgaris*, it was observed that PIPs’ responses to drought stress vary depending on whether the plants is inoculated with arbuscular mycorrhizal fungi or not [71]. Arbuscular mycorrhizal symbiosis with maize plants has been shown to regulate aquaporin expression differentially in short term and prolonged drought stress conditions. Furthermore, various ZmPIPs, specifically the expression of *ZmTIP1;2, ZmTIP2;3*, and *ZmNIP2;1* also varies in response to drought and symbiosis [72].

In transcriptome studies, the roles of PIPs in drought response is well pronounced in most of the cases but some reports specifically point out the role of TIPs in water deficient conditions. In rice, the expression of *OsTIP1;1* was up-regulated in roots and shoot in response to water stress [73]. Root transcriptome analysis of chickpea plants under drought stressed showed both up and down-regulation of different *PIPs, TIPs,* and *NIPs* homologs suggesting their complex integrated roles in regulation of water balance under water deficit [74]. Reports about involvement of NIPs, SIPs, and XIPs in drought stress responses are very few. Detailed transcriptome studies of all aquaporin homologs in citrus plants under drought stress conditions revealed that all *CsTIPs* (11) and *CsXIPS* (3) were up-regulated in leaves but down-regulated in roots, whereas in leaves only two *CsPIPs* (*CsPIP1;1* and *CsPIP2;4*), one *CsNIP* (*CsNIP1;1*), and one *CsSIP (CsSIP1;2*) were differentially up-regulated [13].

On the basis of transcriptome data, it is difficult to identify a concerted pattern of aquaporin expression in response to drought stress. Reverse genetics approaches based on one or a few genes provide a better snapshot than the whole transcriptome studies. Crucial involvement of PIPs in modulating *L*p_r_ in response to various environmental stresses has been widely observed [11,49,75]. Increased susceptibility of plants to water stress due to reduction in *L*p_r_ by silencing PIPs has been reported extensively in various plant species. For example, in Arabidopsis knockout mutants of *AtPIP1;2* and *AtPIP2;2* genes, there was a noteworthy reduction in water permeability of protoplasts [76] and a 14% decrease in *L*p_r_, respectively making these mutants more vulnerable to drought stress [77]. Double antisense lines with reduced expression of *AtPIP1* and *AtPIP2* in Arabidopsis showed up to a 30-fold decrease in *L*p_r_. In tobacco a 55% reduction in *L*p_r_ and increased sensitivity to drought was observed by targeting *NtPIP1* gene using antisense technology. In addition to decreased *L*p_r_, reduced expression of *NtPIP1* also showed a significant decrease in transpiration rate [49]. In moss, *Physcomitrella patens*, knockout mutants of *PpPIP2;1* and *PpPIP2;2* exhibited severe stress phenotypes when grown under water-limited conditions. Based on these observations, it is plausible that these targeted aquaporins play a cumulative role as water transporters, and their decreased expressions make plants vulnerable to water stress due to a decreased *L*p_r_. Further, it is also possible that decreased transpiration rates cause a reduction in photosynthesis, in turn affecting the overall survivability of the plant [78].

Consistently, transgenic plants overexpressing aquaporins showed enhanced drought tolerance. For example, the overexpression of a BnPIP1 from *Brassica napus* in transgenic tobacco plants resulted in increased tolerance to drought stress [79]. Similarly, transgenic tobacco plants overexpressing the wheat aquaporin gene *TaAQP7* (*PIP2*) were more drought tolerant in comparison to non-transgenic tobacco plants due to enhanced water retention capabilities of transgenic plants [80]. Transgenic Arabidopsis plants expressing a *Vicia faba* *PIP1* (*VfPIP1*) showed improved drought resistance by preventing water loss through transpiration due to the induction of stomatal closure [81]. In Arabidopsis, the overexpression of a banana *PIP* gene *MaPIP1;1* showed increased root growth and enhanced survival rates of transgenic plants under drought stress, when compared to wild type plants [82]. Similarly, transgenic banana plants expressing banana *PIP1;2* driven through two diverse promoters conferred enhanced tolerance to drought stress [17]. Overexpression of a tomato *SlTIP2;2* gene in transgenic tomato plants resulted in increased drought-tolerance due to ability of plant to regulate its transpiration rate under drought stress conditions [83]. The above experimental evidence suggests that the overexpression of aquaporins make plants more resistant to drought stress. However, some contrasting results have also been observed because rapid water loss due to increased leaf and root hydraulic conductivity makes some plants even more vulnerable to drought stress conditions. For example, the overexpression of *AtPIP1;4* or *AtPIP2;5* in transgenic Arabidopsis plants showed no advantage under normal conditions but presented a rapid water loss under drought stress conditions. Similarly, overexpression of an Arabidopsis aquaporin gene *AtPIP1b*, in tobacco showed drastic water loss under drought stress [84].

In order to understand the role of aquaporins in drought tolerance many studies have been conducted in naturally drought tolerant plant species. In a drought tolerant plant *Eragrostis nindensis* (resurrection grass), *TIP3;1* was found in small vacuoles of desiccation tolerant leaves, suggesting its importance in enhanced mobilization of water and solutes from these vacuoles upon rehydration [85]. Functional characterization of PIPs in two differential drought responsive poplar clones *Populus balsamifera* and *P. simonii* × *P. balsamifera* revealed a clear correlation between differential expressions of PIP genes in drought avoiding and drought tolerating strategies adopted by these two clones. The rapid reduction of stomatal conductance by reduction in PIPs activities was observed in leaves of drought avoiding *P. simonii* × *P. balsamifera* [86]. Water deficit exposure to a drought tolerant rice cultivar revealed up-regulation of the *OsPIP1;3* gene suggesting its role in drought avoidance [87]. Strong up-regulation of some PIPs in a dehydration tolerant succulent plant, *Craterostigma plantagineum*, was observed under water stress [88]. Characterization of a *FaPIP2;1* gene isolated from a drought-tolerant perennial grass species *Festuca arundinacea* showed its involvement in leaf dehydration status during water stress by overexpressing this gene in Arabidopsis. Transgenic plants showed enhanced resistance and better growth and development under drought stress [89]. In a drought tolerant Vitis hybrid, Richter-110 (*Vitis berlandieri* × *Vitis rupestris*) expression of five PIPs and two TIPs was monitored at different levels of water stress, and it was found that the aquaporin genes in leaves show differential regulation in response to moderate and drastic water stress. A moderate decrease in water availability results in down-regulation of the aquaporins. However, in roots, aquaporin expression showed complex patterns, with no generality among different aquaporins [69].

It can be concluded that drought stress response of aquaporins is highly variable depending on stress levels, aquaporin isoform, tissue, species, presence of symbionts, and the nature of stimuli causing dehydration similar to drought stress. However, a general down-regulation of most of the *PIP* genes is thought to reduce water loss and to help prevent backflow of water to drying soil. Although *TIPs* are found to play a key role in controlling cell water homeostasis by rapid water transport between the vacuole and cytoplasm of plant cells, experimental evidence on their roles in response to drought stress are limited in comparison to *PIPs*.

## 5. Aquaporins in CO_2_ Homeostasis in Water Deficit Conditions

CO_2_ conductivity and transpiration are intrinsically associated with stomatal movement. In order to cut off the loss of water through transpiration, stomatal closure is the paramount water-saving strategy of plants under water-limiting conditions, which result in a reduced rate of photosynthesis due to the unavailability of CO_2_. In addition to their pivotal role in the transmembrane, water transport, and regulation of stomatal aperture, reports have also shown the involvement of *PIPs* in CO_2_ conductivity relevant to the photosynthetic capacity of plant under water-limiting conditions. In tobacco, the involvement of *NtAQP1* has been shown in leaf CO_2_ transport [90]. An *AtPIP1;2* T-DNA insertion line facilitates the diffusion of CO_2_ suggesting a relevance of CO_2_ transport through aquaporins [91,92]. The *Mesembryanthemum* *crystallinum* aquaporin McMIPB, which is classified as a PIP1 that interacts with PIP2 by forming heterotetramers, showed enhanced mesophyll and stomatal conductance, CO_2_ diffusion, photosynthesis, and overall plant growth. This suggests that the co-expression of *PIP2* and *PIP1* may increase the activity of PIP1 not only for water flow but also for CO_2_ diffusion [93]. In Eucalyptus trees, the expression of *EgPIP1* and *EgPIP2* was co-suppressed by using *RsPIP1;1* (*Raphanus sativus* aquaporin) and a low rate of CO_2_ assimilation was observed [94]. In rice, when *HvPIP2;1* is overexpressed, enhanced CO_2_ conductance and CO_2_ assimilation is observed, but there is also greater sensitivity to salt stress [95]. In tomato (*Solanum lycopersicum*), overexpression of *SlTIP2;2* regulates transpiration rates under stress conditions resulting in improved CO_2_ uptake and a more balanced water and nutrient supply [83].

Subsequently, under water stress conditions aquaporins are not only responsible for minimizing water loss from plant tissues, but they also serve a prized function in facilitating CO_2_ homeostasis.

## 6. Roles of Aquaporins in Salt Stress

Soil salinity is the combination of water and ionic stress that exerts noxious effects on plant growth and development by disturbing the cell’s osmotic balance. The primary responses of plants to salt stress are the inhibition of root-water uptake and a resultant decrease in *L*p_r_ [96], which is also manifested in cases of drought stress responses_._ Thus, most of the experimental evidence of aquaporin responses in salinity is in consistent with drought stress. For example, in Arabidopsis a significant reduction in *L*p_r_, coupled with a 60% to 75% decrease in *PIP* and *TIP* aquaporin transcripts abundance, was observed after exposure to salt stress. Transgenic banana plants overexpressing *MusaPIP1;2* and *MusaPIP1;2* displayed enhanced tolerance to both salt and drought stresses [17]. Transcript and protein levels of the barley *HvPIP2;1* gene were found to be down-regulated in roots but up-regulated in the shoots of plants under salt stress [97]. Additionally, the overexpression in transgenic barely revealed increased salt sensitivity, furthering suggesting a relevant role in salt tolerance [98]. A salinity-tolerant ice plant (*M. crystallinum*) also showed down-regulation of many PIP genes [99] and a *TIP* gene (*MIPF*) in roots and leaves, respectively under salt stress [100]. In Maize plants, an ABA mediated, salt-induced down regulation of most of the members of *ZmPIP1* and *ZmPIP2* were observed, but a transiently enhanced expression of *ZmPIP1;1*, *ZmPIP1;5*, and *ZmPIP2;4* was also seen preferentially in the outer parts of the roots. No change in expression of *ZmTIPs* was observed in these experiments [101]. Subsequent studies showed involvement of some TIPs, therefore it can be anticipated that in addition to their role in increased water transport across the tonoplast, TIPs are also involved in the accumulation of ions in vacuoles in response to salt stress. For example, the overexpression of a*AtTIP5;1* in Arabidopsis resulted in the tolerance of transgenic plants to high levels of borate, suggesting its involvement in vacuolar compartmentation of borate [102]. In rice, the expression of *OsTIP1;1* was down-regulated in response to cold stress [12] but up-regulated during response to water and salinity stress [73]. The overexpression of the *Panax ginseng* aquaporin, *PgTIP1*, *in* Arabidopsis showed increased plant growth under optimal conditions and also enhanced tolerance to salt and drought stress [103]. Several studies involving the localization of aquaporins during salt stress have revealed relocalization or redistribution of aquaporins in response to high salt concentrations. For example, salt stress induced re-localization of AtTIP1;1 into intravacuolar invaginations is also shown in Arabidopsis [96]. In addition to redistribution, an alternative mechanism regulating PIP abundance in the plasma membrane is endocytosis of PIPs either through the clathrin-dependent pathway [104] or a salt- stimulated membrane raft–associated pathway [105]. Relocalization of a TIP1;1-GFP into intracellular spherical structures and internalization of PIP2;1-GFP in response to salt stress was also observed in Arabidopsis [96]. Both endocytosis and exocytosis caused by the cycling of AtPIP2;1 to and from the plasma membrane is also observed under salt stress [106].

Similar trends of *PIPs* and *TIPs* regulation in response to salt stress have been observed with only a few exceptions. For example, in drought-tolerant, salinity-sensitive grapevine the expression of the *PIP2;1* gene is up-regulated under salt stress but down-regulated under drought [63]. In addition to PIPs and TIPs, being small solute and ion transporters, NIPs have also been found to be involved in salinity stress. The overexpression of wheat *NIP*, *TaNIP*, in Arabidopsis plants showed increased tolerance to salt stress. Expression of this *TaNIP* gene is up-regulated after ABA treatment, suggesting its association with other ABA regulated pathways [107]. Transcriptome analyses of citrus roots and leaves under salt stress revealed that in addition to *CsPIPs* and *CsTIPs,* most of the *CsNIPs, CsXIPs,* and *CsSIPs* are also up-regulated in roots, whereas in leaves both up and down regulation patterns were observed for some homologs of each aquaporin family except CsSIPs [13]. Similar to drought stress, most of the experimental evidence shows involvement of PIPs and TIPs, while only a few experiments point towards the involvement NIPs. Interestingly, only one account exists of XIPs’ involvement in salt stress.

## 7. Cold Stress and Aquaporins

Similar to water and salt stresses, cold stress is an important abiotic stress factor that significantly limits plant growth and development. The early response of Aquaporins to cold stress has been frequently reported in literature. For example, transgenic banana plants over expressing *MusaPIP1;2* and *MusaPIP1;2* displayed enhanced tolerance to both cold and drought stress [17]. Similarly, transgenic tobacco plants overexpressing a wheat aquaporin *TaAQP7* (PIP2) gene exhibited enhanced cold-tolerance as well as drought tolerant in comparison to non-transgenic tobacco plants [80,108]. Distinctly, other experiments reveal opposite patterns. For example, transgenic Arabidopsis plants overexpressing *AtPIP1;4* or *AtPIP2;5* showed enhanced tolerance to cold stress but are more susceptible to drought due to rapid water loss. In cold sensitive plants like rice, prolonged exposure to cold stress cause an increase in *L*p_r_ which should be regulated through root aquaporins, as most, particularly *OsPIP2;5,* are found to be up-regulated [109]. In another experiment in rice, the mRNA levels of ten genes including *OsTIP1;1* and *OsTIP2;2* were significantly down-regulated but the expression of *OsPIP1;3* increased up to 60% in roots on exposure to chilling treatment [12]. *OsPIP1;3* is also significantly up-regulated in response to drought stress in drought-tolerant rice cultivar [87]. In contrast, *OsTIP1;1* was up-regulated in response to water and salinity stress [73]. While some homologs present similarities in all dehydration inducing stresses like drought, salinity, and cold, others like *OsTIP1;1*, in the following example, are counter-regulated in cold in comparison to drought and salt. *OsPIP1;1* presents different patterns of expression upon exposure to diverse osmotic stresses. Moreover, its involvement in all salt, drought and chilling tolerance has been suggested [64,65].

In Arabidopsis, only *PIP2;5* and *PIP2;6*, which are normally among the low- expressed PIPs, are significantly up regulated by cold stress in both the roots and aerial parts of the plant. All other PIP genes were found to be down regulated by cold stress and their pattern of expression vary with the application of salt or drought stress. Most abiotic stresses, including chilling, induce the production of ABA. Plant responses to stress are generally ABA mediated. However, in an experiment, responses of each aquaporin to ABA treatment were different, suggesting that the regulation of aquaporins might follow both ABA-dependent and ABA-independent signaling pathways [58,67]. Aroca *et al.* in 2005 investigated the effect of long and short term chilling stress on the expression of aquaporins in cold-tolerant and cold-susceptible maize cultivars. They reported that long and short term chilling differentially induce aquaporin responses in similar patterns in both genotypes. These responses lead to recovery in tolerant genotypes, but not in sensitive genotypes including those that are unable to cope with the oxidative stress caused by chilling. Down regulation of *PIPs* in both cultivars is consistent with previous findings in rice and sugarcane [65,110,111].

The response of aquaporins to cold stress is linked with an increase in root hydraulic conductivity that may be regulated through increased aquaporin expression levels in plant roots upon exposure to low temperatures. Differential responses have been observed between short term and long term exposure to cold stress. Moreover, recovery from cold stress has been linked with aquaporin expression, particularly in PIPs.

## 8. Aquaporins in Micronutrient Homeostasis and Heavy Metal Stress

Although the water transport capacity of NIPs is very limited, they play a fundamental role in transporting other substrates involved in various cellular processes. Most *NIP* homologs appear to transport nutrients in plants. Arabidopsis knockout mutant of the *NIP5;1* gene displayed striking growth retardation in response to low supply of boron (B). B is an essential element for plant growth, development, and reproduction, and its deficiency in arable areas has drastically effected crop production worldwide [112]. In the event of high B supply, a feedback inhibition of the *AtNIP5;1* gene is observed, thus providing a strong evidence of the involvement of *NIP5;1* in B homeostasis the adaptation of plants to B toxicity in soil. These results were further confirmed in maize, where a loss of function mutation in *ZmNIP3;1,* a maize ortholog of *AtNIP5;1*, was shown to be responsible for an abnormal phenotype caused by B deficiency. In addition, *AtNIP6;1* and *AtNIP7;1* homologs serve in B transport in Arabidopsis, facilitating its distribution in shoots and anthers, respectively [113,114]. Tolerance to high B was observed in barley by a reduced expression of the *HvNIP2;1* gene. [115]. In addition to *NIPs*, an overexpression of *TIP5;1* in Arabidopsis suggested its involvement in vacuolar compartmentation of borate [102]. Silicon (Si) plays an important role in plant defense against biotic and abiotic stresses. Si-mediated alleviation of plant stress is a well-known phenomenon [116]. The Si transporter *OsNIP2;1* (Lsi1) was identified by quantitative trait loci (QTL) mapping in a rice mutant defective in Si uptake [117]. Furthermore, the Si transporter *NIP2;2* (Lsi6) was also identified from rice [116]. Therefore, OsNIPs seems to play a significant role in Si transport in rice plants.

In addition to nutrient transport, NIPs are also found to be involved in both the transport and responses to heavy metal stress. A T-DNA knockout mutant of Arabidopsis *NIP1;1* showed arsenite (As) tolerance suggesting its role as an As transporter [118]. Recently, *AtNIP3;1* has shown to be involved in As transport. Double knockout mutants for both *AtNIP1;1* and *AtNIP3;1* show more pronounced tolerance against As stress. An additional four other isoforms, *NIP5;1*, *NIP6;1*, *NIP7;1*, and *NIP1;2*, are reported to be capable of As(III) transport on the basis of their expressions in yeast and oocytes [119]. The transport of antimony (Sb) by *AtNIP1;1* has also been established, and its loss of function mutant showed an increased tolerance to high Sb stress [120]. A lactic acid transporter *AtNIP2;1* was shown to be associated with water logging, leading to oxygen deprivation. Thus it can be anticipated that *AtNIP2;1* is an anaerobic-induced lactic acid transporter that may play a role in plant adaptation to lactic fermentation under anaerobic stress conditions [121].

## 9. Aquaporins in Plant Symbiotic and Pathogenic Relations

Plant symbiotic relationships are known to affect plant aquaporin responses under different abiotic stress conditions [71,72]. It is hypothesized that in plant symbiotic relations, the re-distribution of water and nutrients takes place between the host and symbionts further shedding light on a relationship with aquaporins. The roles of NIPs in plant interactions with symbionts and pathogens are obvious but experimental evidence is scarce. The first identified aquaporin, nodulin-26, was found in soybean root nodules and is speculated to have formed as a result of a symbiotic interaction between plant and nitrogen fixing rhizobium. While the roles of NIPs in various forms of nitrogen transport (urea, ammonia) are well known, the mechanistic involvement of NIPs in driving these nutrient exchanges between plants and symbionts or plants and pathogens needs further investigation. The roles of plant aquaporin-mediated solute transport during plant symbiosis with arbuscular mycorrhizae have been examined by gene profiling. Such results have indicated that the transport of glycerol from plant to the microbe, in addition toNH_4_/NH_3_ from microbe to plant, is mediated by aquaporin genes including *NIPs* [72].

Like symbionts, plant pathogens rely on plant nutrients for their growth and survival on their plant hosts. Dehydration, as a result of pathogen infection, is a common observation responsible for regulating plant water relations and nutrient homeostasis. The production of reactive oxygen species is an integral component of plant immune system. The transport of H_2_O_2_ by aquaporin homologs also highlights functions in plant defense, despite not having a full understanding of these pathways [122,123]. Expression profiling of soybean leaves upon *Pseudomonas syringae* infection revealed down-regulation in 24 of 32 soybean aquaporin genes [124]. In citrus plants, six CsMIPs (*CsPIP1;2, CsPIP2;2, CsNIP2;2, CsNIP5;2, CsNIP6;1*, and *CsSIP1;1*) were found to be differentially expressed exclusively under the biotic stress imposed by the citrus infecting proteobacterium, *Candidatus Liberibacter*. Comparisons of *CsPIP2;2*, *CsTIP1;2*, *CsTIP2;1*, *CsTIP2;2*, and *CsNIP5;1* expression patterns of susceptible sweet orange and tolerant rough lemon cultivars revealed that most *CsMIPs* are down regulated. Thus, they could be correlated with the disease development [13,125]. As a result, the roles of aquaporins in response to pathogen infection are highly anticipated. Evidence of NIPs’ involvement in biotic stress responses in maize was reported [126]. The induction of a tobacco root specific *TIP1* gene expression upon nematode infection was observed [127]. In grasses (Festuca spp.), Si accumulation offers an effective defense against herbivore attacks [128]. Thus, the regulation of Si uptake through NIPs can be correlated with plant defense against herbivory. The precise modes of aquaporin regulation during infection and their response to pathogen signals are still unknown. Some interactions of aquaporins with bacterial and oomycete effectors were observed in yeast two hybrid systems [129]. TIP1 and TIP2 were found to interact with a cucumber mosaic virus (CMV) replication protein, CMV1a, in the SOS recruitment system suggesting that this TIPs-CMV1a interaction potentially affects CMV replication in the host plant’s tonoplasts [130]. Interactome and transcriptome data provide important clues about the involvement of aquaporins in host-pathogen interactions. Extensive reverse genetic-based characterization of individual or multiple putative aquaporin homologs needs to be undertaken in relation to pathogen infection to reach a definite conclusion about role of aquaporins in plant diseases.

## 10. Complex Integrated Roles of Aquaporins in Plant Stresses

**Figure 3:**
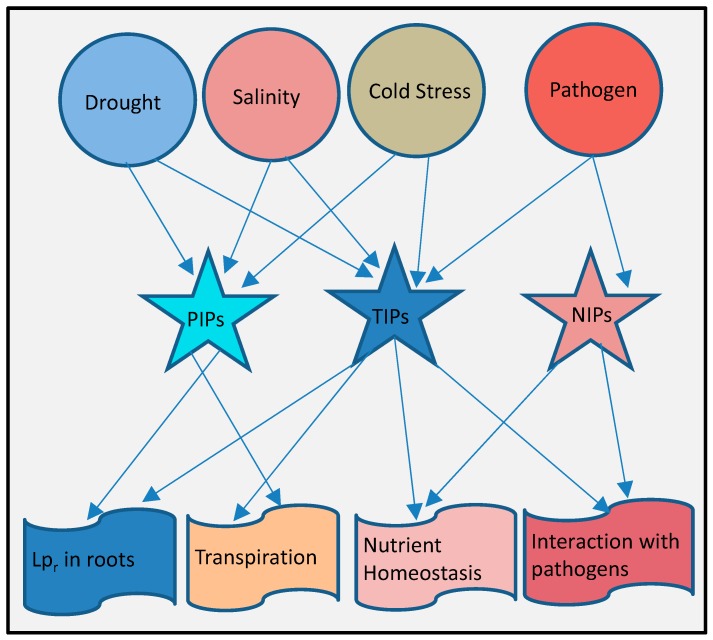
A generalized summary of plant aquaporins in response to environmental stimuli. Both PIPs and TIPs are more responsive to drought, salt and cold stress that disturb cell osmotic balance, they regulate root hydraulic conductivity (*L*p_r_) and transpiration rates. TIPs along with NIPs are involved in biotic stress responses and involved in regulating nutrient homeostasis between host and its pathogen. NIPs and TIPs are also found to interact with some pathogen proteins.

Despite enormous experimental evidence about the involvement of aquaporins genes in various stress responses (Figure 3), precise roles of individual genes or particular subfamilies are still hard to define because of their highly complex and integrated roles in the response to different environmental stimuli and involvement in other plant growth and developmental processes. These integrated contributions in multiple physiological facets can be more pronounced in transgenic plants containing foreign aquaporin genes. In transgenic plants expressing an exogenous aquaporin gene, tolerance or susceptibility is collectively effects by interactions with other plant genes. Transgenic Arabidopsis plants expressing a *Vicia faba* *PIP1* (*VfPIP1*) showed improved drought resistance due to induction of stomatal closure, thereby preventing water loss in water- deficient conditions [81]. Hence, the interaction of *VfPIP1* with other genes and gene products involved in stomatal movement contributes to overall drought tolerance. The silencing of endogenous genes by expressing exogenous aquaporins has also been observed when the *Raphanus sativus* aquaporin *RsPIP1;1* gene is expressed in eucalyptus causing the silencing of endogenous *EgPIP1* and *EgPIP2* PIP aquaporins [94]. The overexpression of *AtPIP1b* in tobacco results in an increased stomatal density and increased growth rates under favorable conditions but drastic water loss under drought stress [84]. Transgenic tobacco plants overexpressing a wheat aquaporin *TaAQP7* (*PIP2*) gene showed increased activity of detoxification enzymes resulting in an improved antioxidant defense and a reduction in H_2_O_2_ levels under osmotic stress [80]. Similarly, the overexpression of a wheat *PIP*, *TdPIP1;1*, in tobacco showed an enhanced stress tolerance coupled with increased leaf area and increased root size [131]. The overexpression of two foreign aquaporin isoforms, *CfPIP2;1* (*Cucurbita ficifolia*) and *CsPIP1;1* (*C. sativus*) in Arabidopsis, noticeably perturb the natural expression of other aquaporins, resulting in altered stress responses of the plants under various stress conditions [132]. In addition, the overexpression of a *BnPIP1* from *Brassica napus* in transgenic tobacco plants results in an increased tolerance to drought stress, increased plant growth, and higher seed germination rates, whereas antisense plants showed developmental deformities and an increased susceptibility to drought stress [79].

In addition to these outcomes of over-expressing aquaporins in different transgenic backgrounds, advances in interactomics has shown physical interactions among different aquaporin homologs [133,134,135] as well as aquaporin interactions with several other proteins involved in various diverse functions in plants, alluding to their complex, integrated role in different physiological and stress responses [130,136,137,138,139,140,141].

## 11. Conclusions

Current data suggest that drought and salt stress induces more dramatic changes in aquaporin expression than any other abiotic stress [19]. A profound role of PIPs in response to osmotic stress stimuli like drought and salinity can be established (Figure 3). Knockout mutants of *PIPs* and their overexpressing transgenic plants revealed that PIPs respond to osmotic stresses in an integrated, complex way by regulating root water uptake and increasing transpiration rates as well as affecting the overall plant growth and development processes. Like PIPs, TIPs are involved in regulating osmotic stress responses, but experimental evidence is far less in comparison to PIPs. TIPs are also found to be involved in the uptake of some micronutrients facilitating responses to higher micronutrient concentrations. NIPs’ roles in nutrient homeostasis and heavy metal stress responses are more pronounced than any other aquaporin subfamily. Information on the involvement of aquaporins in biotic stress responses is still scarce. However, current analyses on the involvement of TIPs and NIPs in nutrient uptake points to a pronounced involvement of NIPs in plant pathogen interactions (Figure 3).

Since the discovery of aquaporins two decades ago, more information has been unveiled regarding structure, substrate specificity, gating properties, subcellular localization, and roles in various physiological processes in plant growth and development. Forward and reverse genetic approaches have also been used extensively to define their roles in plant defense mechanisms against environmental stresses. However, a clear cut mechanistic role of individual aquaporin homologs in response to a particular environmental stress has not yet been established because of the complex, integrated responses that vary among different plant species. Numerous studies of plant aquaporins under osmotic stress conditions have revealed their importance in regulating plant stress responses. Rapid changes in the expression levels of aquaporins, in response to diverse stresses, have been documented often, but the molecular and cellular mechanisms underlying these responses are still unknown. The localization patterns in specific membranes and subcellular compartments combined with their redistribution with exposure to diverse osmotic stresses could be crucial for efficacy of aquaporins [142,143]. Moreover, the responses of aquaporins to various stress induced hormones indicate their involvement in stress induced hormonal pathways. The interactions of aquaporins with other plant proteins also provide an insight into the complex mechanisms involved in aquaporin regulations and diverse stress responses. In order to arrive at a definite conclusion, more detailed studies are needed to map different pathways of aquaporin activities in plant physiology and stress responses. Differential responses of aquaporin homologs to stress induced hormones need thorough investigation to uncover their mechanistic involvement in plant stress responses.

## Abbreviations

The following abbreviations are used in this manuscript:

*L*p_r_: Root hydraulic Conductivity
MIPs: Major intrinsic proteins
*MIPs*: Major intrinsic protein genes
PIPs: Plasma membrane intrinsic proteins
*PIPs*: Plasma membrane intrinsic protein genes
TIPs: Tonoplast intrinsic proteins
*TIPs*: Tonoplast intrinsic protein genes
NIPs: Nodulin-26 like intrinsic proteins
*NIPs*: Nodulin-26 like intrinsic protein genes
SIPs: Small basic intrinsic proteins
*SIPs*: Small basic intrinsic protein genes
GIPs: GlpF-like intrinsic proteins
HIPs: Hybrid intrinsic proteins
AQPs: Aquaporins
XIPs: Uncategorized X intrinsic proteins
*XIPs*: Uncategorized X intrinsic protein genes
ROS: Reactive oxygen species
GLPs: Glycerol-facilitators
ER: Endoplasmic reticulum
ΔΨ: Water potential
CMV: Cucumber mosaic virus
